# Merging microarray data, robust feature selection, and predicting prognosis in prostate cancer

**Published:** 2007-02-14

**Authors:** Jing Wang, Kim Anh Do, Sijin Wen, Spyros Tsavachidis, Timothy J. McDonnell, Christopher J. Logothetis, Kevin R. Coombes

**Affiliations:** 1 Department of Biostatistics and Applied Mathematics,; 2 Department of Molecular Pathology,; 3 Department of Genitourinary Medical Oncology, The University of Texas M. D. Anderson Cancer Center, Houston, TX, USA.

**Keywords:** combining data, cross-validation, feature selection, microarray expression profiling, predictive model, prostrate cancer

## Abstract

**Motivation:**

Individual microarray studies searching for prognostic biomarkers often have few samples and low statistical power; however, publicly accessible data sets make it possible to combine data across studies.

**Method:**

We present a novel approach for combining microarray data across institutions and platforms. We introduce a new algorithm, robust greedy feature selection (RGFS), to select predictive genes.

**Results:**

We combined two prostate cancer microarray data sets, confirmed the appropriateness of the approach with the Kolmogorov-Smirnov goodness-of-fit test, and built several predictive models. The best logistic regression model with stepwise forward selection used 7 genes and had a misclassification rate of 31%. Models that combined LDA with different feature selection algorithms had misclassification rates between 19% and 33%, and the sets of genes in the models varied substantially during cross-validation. When we combined RGFS with LDA, the best model used two genes and had a misclassification rate of 15%.

**Availability:**

Affymetrix U95Av2 array data are available at http://www.broad.mit.edu/cgi-bin/cancer/datasets.cgi. The cDNA microarray data are available through the Stanford Microarray Database (http://cmgm.stanford.edu/pbrown/). GeneLink software is freely available at http://bioinformatics.mdanderson.org/GeneLink/. DNA-Chip Analyzer software is publicly available at http://biosun1.harvard.edu/complab/dchip/.

## Introduction

Prostate cancer, the second leading cause of cancer death in men in the United States, exhibits a broad range of clinical and histological heterogeneity, and its diagnosis and prognosis depend substantially on tumor behavior at the molecular level. Microarray technology has helped to identify tumor subtypes, and to search for diagnostic and prognostic biomarkers (Magee et al. 2001; Welsh et al. 2001; Singh et al. 2002; Lapointe et al. 2004).

Individual microarray investigations typically involve few clinical samples, and therefore have low statistical power to identify predictive genes. The growing availability of public data sets facilitates combining data across studies and laboratories, which can increase the sample size and thus the power to detect true expression results with fewer false positives. Such meta-analyses are critically important for the further development of this technology as a useful clinical tool.

Several investigators have performed meta-analysis on microarray data in cancer research (Rhodes et al. 2002 and 2004; Ghosh et al. 2003; Choi et al. 2004; Wang et al. 2004); however, significant analytical challenges to this process remain. They include a lack of accepted data standardization procedures; substantial experimental variability; incomparable numerical scales of expression measurements across platforms; and the need for associated clinical and pathological information for each microarray data set, which is not always available.

We developed a novel approach for combining tumor gene expression measurements across microarray platforms, and evaluated its usefulness for biomarker discovery. Our objectives were to correlate gene expression measurements with prognostic indices, and to construct probabilistic models in order to reveal genes that significantly separated patients with good prognoses from patients with poor prognoses.

Feature selection is a critical step for building models to predict outcomes or classify samples based on high dimensional data, such as microarray gene expression measurements. The goal is to select a subset of features that is optimal for classification or prediction. Although it is reasonable to believe that a small set of genes contains enough information to distinguish clinically relevant categories, the practical question of how best to select an informative set of features from large-scale expression data remains open.

A wide variety of feature selection algorithms have been proposed (Guyon and Elisseeff, 2003; Pochet et al. 2004; Inza et al. 2004; Hauskrecht et al. 2005; Choudhary et al. 2006). The algorithms include ‘filters’ that select features based on univariate rankings (Golub et al. 1999; Chow et al. 2001; Dudoit et al. 2003), data reduction methods such as principal component analysis (West et al. 2001; Tan et al. 2005); and ‘wrappers’ like genetic algorithms (Ooi et al. 2003; Deutsch 2003; Baggerly, et al. 2003) that attempt to select multiple features simultaneously based on their performance on a training set. Although the results are encouraging, there is still no convincing evidence to show that any particular algorithm always performs better.

The fundamental difficulty lies in the tendency to over-fit the data–to select features that are accidental artifacts of a single data set–when the number of features is far greater than the number of samples to be classified. Consequently, any proposed method of feature selection and classification must be validated either on a completely independent data set or by cross-validation (repeatedly splitting the initial data set into subsets for training and testing). For proper cross-validation, feature selection must be performed separately during each iteration, only using information from the current training set. If, instead, one selects features using the entire data set and only cross-validates the step that optimizes the parameters in the classifier, then one gets an overly optimistic estimate of the classification accuracy (Ambroise and Maclachlan, 2003; Simon et al. 2003B).

In this study, we applied several algorithms for feature selection to a combined prostate cancer array data set. The performance of all algorithms was evaluated using leave-one-out cross-validation, with the feature selection step performed inside the cross-validation loop. In particular, we introduced a new approach, greedy robust feature selection (GRFS), and applied it to the combined data. GRFS is a wrapper method for feature selection that relies on another leave-one-out loop (inside the cross-validation loop) to identify a robust feature set. The usefulness of GRFS, as well as the issues involved in feature selection and cross-validation, will be discussed.

## Materials and methods

We used two publicly available microarray gene expression data sets, which we refer to as the Harvard and the Stanford data set. These data were collected using two distinct platforms: Affymetrix GeneChip^®^ U95A and two-color florescent-labeled glass microarrays (Table 1).

The Harvard data set was obtained through a study of the correlation of gene expression and clinical behavior for prostate cancer (Singh et al. 2002). The data include 102 Affymetrix U95Av2 GeneChips from 50 healthy prostate samples and 52 prostate tumor samples, consisting of 12,625 probe sets representing 7,865 distinct UniGene clusters (UniGene Build 170). It is available at the Center for Genome Research, Whitehead Institute (http://www.broad.mit.edu/cgi-bin/cancer/datas-ets.cgi).

The Stanford, two-color fluorescent cDNA microarray data were obtained through a study identifying clinically relevant subtypes of prostate cancer (Lapointe et al. 2004). The set consists of data from 41 healthy prostate specimens, 62 primary prostate tumors, and 9 unmatched lymph node metastases. The microarrays contain 42,129 spots for 38,804 different cDNA clones representing 21,287 distinct UniGene clusters (UniGene Build 170). The prostate cancer samples were labeled with Cy5. A common reference material, pooled from 11 established human cell lines, was labeled with Cy3. The raw micro-array data can be downloaded from the Stanford Microarray Database (http://cmgm.stanford.edu/pbrown/).

## Prostate cancer prognostic indices

Prostate tumor prognosis is indicated by the Gleason score, ranging from 2 to 10, which is based on the architectural pattern of the prostate tumor, and indicates its aggressiveness (Gleason and Mellinger, 1974; Gleason, 1977). Clinical studies have shown that the Gleason score correlates with tumor prognosis: a Gleason score of 6 or lower is associated with stable disease and a good outcome; 8 or higher is associated with a bad outcome and the need for additional treatment; and a score of 7 is associated with extremely variable tumor behavior. Determining the molecular signature associated with each Gleason score and predicting the clinical course of prostate cancer remains an important research challenge.

Gleason scores ranging from 5 to 9 are available for tumor samples in both Stanford and Harvard data sets. To make the investigation clinically relevant, we divided the Gleason scores into three categories: low (6 or lower), medium (7; 3+4 and 4+3), and high (8 or higher). The patient populations with tumors categorized into the three groups are summarized in Table 2.

## Processing and combining data

For the Affymetrix GeneChip data, we used DNA-Chip Analyzer (dChip), version 1.3 (Li and Wong, 2001), to obtain gene expression values based on a model that only used perfect match (PM) features. We used dChip to normalize to the array with median brightness, and rescaled the normalized data so that the 75^th^ percentile equaled 1000.

We performed intensity-dependent loess normalization on each cDNA microarray in order to remove intensity-dependent dye bias (Dudoit et al. 2002; Yang et al. 2002), and then normalized the background-corrected spot intensities of each channel so that the 75^th^ percentile equaled 1000. We log transformed (base 2) both array data sets for further analysis.

We used the following standardization procedure for each gene expression level based on the mean expression measurements of, and the standard deviation estimated from, the arrays of healthy prostate tissue:

(2.1) $$g_{i,S}=\frac{{\text{X}}_{i,S}-{\text{X}}_{i,(healthy)}}{{\text{V}}_{i,(healthy)}}$$

Here *g_i,S_* denotes the standardized expression measurements of gene *i* in sample *S; X_i,S_* is the (log transformed) expression level of gene *i* in sample *S* before being standardized; X_*i*, (*healthy*)_ is the mean expression of gene *i* across all healthy prostate samples; and v_*i*,(*healthy*)_ is the standard deviation (SD) of gene *i* computed across all arrays of healthy prostate samples. On each platform, standardized expression measurements from healthy prostate samples should be normally distributed with mean = 0 and SD = 1.

After standardization, the transcripts that have multiple clones on a two-color glass array or multiple probe sets on an Affymetrix array for the same gene need to be combined. We combined these measurements by averaging. Since averaging changes the standard deviation slightly, we re-standardized each data set. This process simply adjusted by the square root of the number of clones or probe sets.

We applied UniGene matching for the critical step of determining which genes had been included on all platforms. For each platform, we assumed that the array manufacturer’s GenBank accession numbers were accurate. Because GenBank numbers identify sequences rather than genes, we replaced them with identifiers linked to the concept of an individual gene: UniGene cluster identifiers. To update the gene annotations to a recent UniGene build (Build 170, July 2004), we used in-house software, GeneLink, which is freely available online (http://bioinformatics.mdanderson.org/GeneLink/). We then combined the array data from genes that were common to both platforms, and obtained a data set consisting of the standardized measurements of 6402 genes from 114 patients with prostate cancer.

Classification models were constructed using the implementations of logistic regression and linear discriminant analysis (LDA) in S-PLUS^®^ (Insightful Corp., Seattle, WA).

## Feature selection

Technically, performing LDA requires computing and inverting the covariance matrix, which requires the number of genes used for prediction to be less than the number of samples used for the investigation. Feature selection is thus the critical step when applying LDA to microarray data sets. We explored several algorithms for feature selection.

A ***greedy algorithm*** is a common method for finding an approximate optimal solution to a problem. The algorithm extends a small part of the data structure by adding locally optimal components one by one, until it achieves an optimal global solution. Forward stepwise selection in logistic regression is an example of a greedy algorithm.

We considered a set of genes to be optimal if it maximized the Mahalanobis distance between the two groups. To perform the greedy algorithm, we started with the gene that had the lowest p-value for a two-sample t-test. We then added one gene at time to minimize the Mahalanobis distance, and computed the classification error from the LDA. This selection process stopped when a perfect classification result was reached or when the process stopped improving the classification error.

A ***genetic algorithm (GA)*** *is* a heuristic search process based on natural genetic selection. Briefly, a string (as an individual) containing encoded parameters (attributes) of the solution is used to represent each potential solution. A set of strings forms a population, and a pool of random strings initializes the population. A fitness function (here, the Mahalanobis distance) determines the degree of fitness of each string. Genetic operators (reproduction, crossover and mutation) are applied to the strings in the pool, creating a new population of strings for the next generation. This process is repeated until a termination condition, such as a maximum number of generations to be run, is satisfied.

***Principal components analysis (PCA)*** attempts to find a set of orthogonal principal components to preserve as much variance as possible in independent variables. This algorithm projects the observations (specimen) from a high-dimensional variable space (gene expression) to a low-dimensional subspace, so that the prediction is feasible. PCA feature selection uses principal components instead of individual genes to perform classification and prediction, selecting the first few principal components, which explain the most variance (Khan et al, 2001; West et al. 2001).

We introduce a ***greedy robust feature selection*** *(****GRFS****)* approach built on the Leave-one-out cross validation (LOOCV) procedure to keep the most frequently identified features for building a predictive model. Leaving out one sample at a time, we used a greedy-LDA to identify a set of predictive genes. We counted the number of times a gene was selected, and retained only the most frequently identified genes as the selected features.

## Results

**Figure 1.**
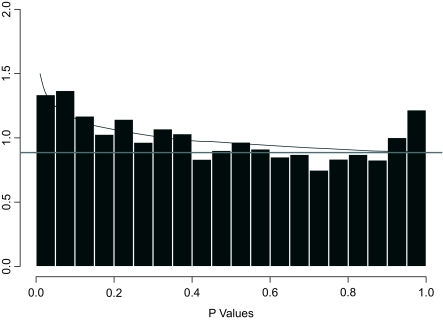
Distribution of p-value computed from Komogorov-Smirnov (KS) goodness-of-fit test to the expression profiles from healthy prostate samples re-standardized on a gene-by-gene basis. The plot illustrates the p-values distributed near uniformly. The superimposed curves represent the division into uniform and β contributions.

The assumption of our standardization procedure is that the expression profiles from healthy prostate should be distributed similarly across array platforms, with the differences in the means and standard deviations resulting from differences in array technology. To validate this assumption, we applied a two-sample Kolmogorov-Smirnov (KS) goodness-of-fit test to the re-standardized healthy population expression profiles on a gene-by-gene basis. The null hypothesis of the KS test is that the expression distributions of each gene from healthy prostate tissues on both platforms have identical cumulative distributions. To adjust for multiple testing, the resulting p-values were analyzed using a beta-uniform mixture (BUM) model (Pounds and Morris, 2003). Under the null hypothesis, we expected to see a uniform distribution of the resulting p-values from the KS test. The p-value distribution is plotted in Figure 1. The plot indicates that the p-values were distributed nearly uniformly, with a small population (about 7%) satisfying *p* < 0.05. Therefore, the assumption for standardizing gene expression profiles appears to be statistically valid.

**Figure 2.**
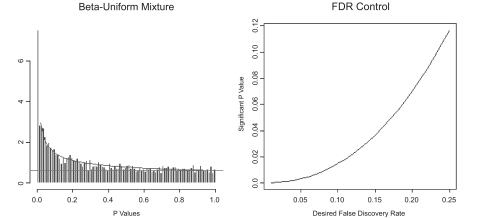
Analysis of *p*-values as a beta-uniform mixture. **Left:** Histogram of the *p*-values from a chi-squared test of the quality of the fit when adding a single gene to the logistic model. **Right:** The plot describing the relationship between FDR and single-test *p*-values.

We built a logistic regression model to predict Gleason score (low or high) in two steps: (i) identification of candidate genes and (ii) forward stepwise variable selection. We fit the logistic regression model in S-PLUS^®^ gene-by-gene, and computed the regression p-value for each gene. We found the study effect in the model to be statistically insignificant. Adjusting for multiple testing, we applied BUM to model the resulting p-values, and assessed the false discovery rate (FDR) to identify significant genes (Benjamini et al. 1995) (see Figure 2).

To build an effective model, we applied forward stepwise variable selection to find an optimal set of predictive genes from the top 20 genes (sorted by p-values) identified by logistic regression, adding genes to the model one at time. Using S-PLUS^®^, we selected a gene at each stage based on the value determined by the likelihood function of the model. From the top 20 genes, we found a 7-gene predictive model that provided perfect classification for our combined data set. These seven genes are LTBP2, TIMP2, CDH11, RAP140, CXCR4, ProSAPiP2, and SEPT6.

**Figure 3.**
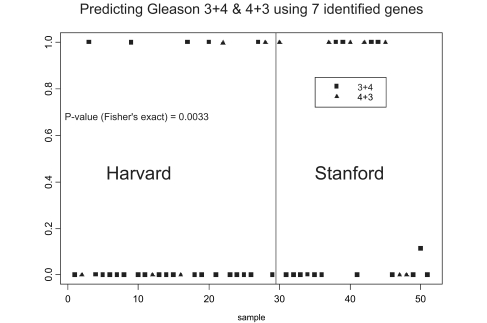
Predicting Gleason Score 7 in an independent test set using the 7-gene model. The data set consists of 6204 gene expression measurements and data from 52 prostate tumors. The squares in the plot indicate Gleason scores 3+4 and the triangles represent Gleason scores 4+3. The results are statistically significant with p=0.033 (Fisher's exact test).

Since we did not have an independent testing set that contained low and high Gleason scores, we applied the 7-gene model to a data set that contained data from prostate tissue given a Gleason score of 7, and which was part of the combined data with the expression measurements from 6402 genes and 52 patients. The model separated them into good or poor prognostic categories that were associated with Gleason grades 3+4 or 4+3, respectively (Fisher exact test; *p =* 0.033). Figure 3 illustrates the findings.

We performed LOOCV to validate our logistic regression model. To properly validate the model, the selection of top genes and forward stepwise variable selection were built into each LOOCV (Simon, 2003B). The misclassification rate by LOOCV was 31%. Of 43 tumors with low Gleason scores and 19 with high scores, the model correctly predicted 33 and 10, respectively. The number of predictors selected varied (from 4–9 genes), and predictors were inconsistent across each leave-one-out training set.

Because the cross-validated misclassification rate was higher than we expected (31%), we evaluated other methods for building classifiers from this data set. We built a prediction model from a larger set of candidate genes identified by logistic regression, first identifying 231 genes based on FDR = 5% (*p* ≤ 0.0032), and then applying a forward stepwise variable selection procedure. We found a 4-gene model that provided perfect classification for our combined data set: LTBP2, JAG1, ASTN and PORIMIN. The misclassification rate by LOOCV was 26%. Again, the number of predictors selected varied (from 3–5 genes), and genes selected as predictors were inconsistent across each leave-one-out training set.

To build the LDA model, we first selected a subset of candidate genes based on a univariate two-sample t-test between the groups with low and high Gleason scores. We computed the p-value of each gene according to the test statistic, and then used BUM to adjust for multiple testing. To identify significant differentially expressed genes between the two groups, we set FDR = 10%. With this criterion, the subset contained about 200–300 genes, depending on the leave-one-out training set. Table 3 summarizes the results of the cross-validation for each model we tested.

From the selected subset of candidate genes, we searched for a small set of genes that achieved the best discrimination by the greedy algorithm. In order to correctly validate the model, we built the selection of candidate genes and feature selection into the LOOCV, i.e., for each leave-one-out training set, we repeated the two-sample t-test and greedy feature selection. The gene with the smallest p-value from the t-test was chosen as the first gene for the model. From each training set, the greedy algorithm selected about 10–20 features that achieved perfect classification. However, the LOOCV misclassification rate was 31%, and the features selected from each leave-one-out training set were inconsistent.

We next applied GA to search for the optimal set of 10 features that gave the best separation of the two Gleason groups (low and high). For each feature N (N = 1,2, ... 10), we performed GA using different randomly generated initial populations, each containing 200 sets of N genes. The GA was allowed to evolve for 500 generations; each run of the GA converged. We chose the set of N genes with maximum fitness as the selected features to perform LDA. For LOOCV, we ran the GA on each leave-one-out training set to identify the 10 “best” genes, and then performed the LDA. The LOOCV misclassification rate was 28%. The model performed poorly in predicting patients with high Gleason scores, and genes selected as features from each leave-one-out training set were inconsistent.

We performed PCA on the selected candidate genes. Keeping enough components to explain 80% of the variance, we then used these components as predictors to perform the LDA. To explain 80% of the variance, the PCA-LDA model selected about 10–20 components and achieved perfect classification on the training sets. The LOOCV misclassification error was 24%.

We applied the GRFS-LDA approach. In this two-level LOOCV feature selection process, we retained the most frequently identified genes from the second level. More precisely, after leaving one sample out, we applied GRFS using another level of leave-one-out on the training set. We retained features that were selected more than 20 times (out of 60) in the second level leave-one-out, and used those features to train an LDA model and to predict the status of the left-out sample at the first level. The model selected 5–11 features, and the LOOCV misclassification rate was 31%.

## Simulation and permutation studies

The LOOCV results suggested that all 5 methods overfit the training set. To understand this problem, we performed a simulation study and two permutation studies. We first simulated 20 data sets containing 6200 variables (genes) and two groups, with 40 and 20 randomly selected samples on each group. The data sets were similar to our training set in dimensions (variable number and sample size), and were simulated as independent, identically distributed, with standard normal noise. We applied a greedy algorithm for feature selection, followed by LDA. In each simulation, between 8 and 11 genes were enough to achieve perfect classification, even though there was no class structure.

We then performed two permutation studies using the combined prostate cancer data set. In the first, we permuted the sample labels 50 times, selecting the top 200 genes by a two-sample t-test, followed by the greedy-LDA. The model achieved perfect classification on the training set with about 20 genes (11–27 on all 50 permutations). In the second, we permuted the sample labels 10 times and used all 6204 genes with greedy-LDA. With the selection of about 13 genes on average (range 9–15), the model achieved perfect classification on the training set.

The results suggested that these models overfit the training data by selecting too many features. We then tested using fewer features (< 8) to build the LDA model (see Table 4). Unexpectedly, the performance of three of the feature selection methods (Greedy, GA, and PCA) showed no significant improvement. However, using only two features, the GRFS-LDAmodel achieved the best classification results with a LOOCV misclassification rate of 15%. The two genes selected by GRFS-LDA on the full data set are latent transforming growth factor beta binding protein 2 (LTBP2; Hs.512776) and pro-oncosis receptor inducing membrane injury gene (PORIMIN; Hs.503709). These two genes were included in the 4-gene logistic regression model.

**Figure 4.**
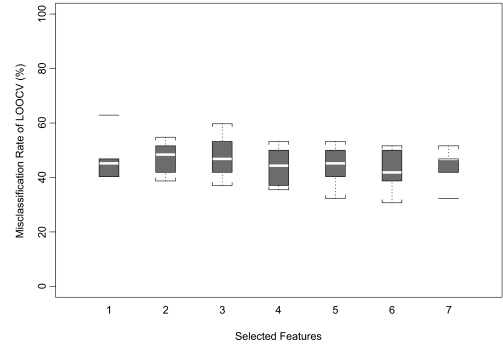
Misclassification rates of leave-one-out cross validation obtained by performing robust feature selection approach on randomly generated data sets (n=10). For seven selected features, the median values range from 41.85 to 48.40.

Finally, we wanted to test whether an overall LOOCV accuracy of 15% was likely to be better than chance on a data set of this size. To test this, we applied the GRFS-LDA algorithm to multiple (*n=*10) simulated non-informative data sets with the same dimensions as the combined prostate cancer data set. Each simulation generated independent, identically distributed measurements. The random data sets were normally distributed with the same mean and standard deviation as the combined prostate cancer data. We repeated the full analysis selecting fewer than 8 features. The results showed that misclassification rates on random data sets were much higher, typically on the order of 40% or more (Figure 4), which is consistent with the fact that these data sets actually have no class structure. One should also note that LOOCV misclassification rates of 30% appear not to be unlikely with 5 or more features.

## Discussion

We have introduced a standardization procedure to combine cancer array data across institutions. Using the Kolmogorov-Smirnov test, we confirmed that the distributions of gene expression from healthy prostate tissue were essentially the same after this procedure, allowing us to combine the prostate cancer data.

We have also examined whether the combined data can be used to identify clinical relevant prognostic markers for prostate cancer. We chose to apply two widely used statistical approaches, since we believe most classification methods should perform similarly once the appropriate predictive genes have been selected. The comparative study by Dudoit and Fridlyand (2003) supports this idea.

We did not intend to compare prediction methods to determine which one performed better. Instead, we focused primarily on combining expression data and on feature selection.

Feature selection appears to be a major challenge in building an accurate predictive model, and is complicated by large expression data sets with substantial measurement noise. No one has yet reported convincing results with proper validation. Michiels and colleagues recently re-analyzed seven published microarray cancer data sets, observing that prediction models based on (containing about 50 genes) predicting cancer prognosis were highly unstable and in their analysis depended strongly on the selection of patients in the training set (Michiels et al. 2005). Our simulation and permutation studies show that it is possible to achieve “perfect” classifications on a training set using a small number of “noise” genes, as few as 10 to 20. These results suggest that overfitting by selecting too many features remains a major problem for the types of models we have studied, and that an independent validation test set is required.

Although the accuracy of a predictive model may appear excellent when it is tested on the same data set from which it was derived, it may perform poorly when applied to a new data set. This is the well-known overfitting problem, which occurs when too many parameters are fit to a small number of data points, resulting in parameters being fit to random noise (Simon et al. 2003A, 2003B). Michiels and colleagues also observed that the misclassification rate estimated by cross-validation was typically much larger than the misclassification rates that had been reported in the original papers without cross-validation (Michiels et al. 2005).

LOOCV is commonly used for validating a method to build predictive models. Since feature selection is an important part of model development, to properly validate a model, the feature selection process must be built into the LOOCV. That is, for each leave-one-out training set, we must repeat the feature selection process; otherwise, we would obtain a biased estimate of the accuracy of the model (Ambroise and Maclachlan, 2003; Simon et al. 2003B). When applying LOOCV to the combined prostate cancer data set, we found that (1) the LOOCV misclassification rates were on the order of 25–30%, and (2) the selected features varied from one training set to another and depended strongly on which samples were used for training.

We hypothesized that the instability in feature selection might be responsible for the high misclassification rate. To avoid this problem and select more consistent features, we developed the GRFS algorithm. This approach attempts to use leave-one-out methods to select a set of genes that should be more stable. The robust approach, however, did not immediately improve the misclassification rate as we expected. In our first application of the method, using a criterion that retained all features that were found in one third of the individual models, we selected between 5 and 11 features. Since our simulations suggested that we could find between 8 and 11 features to classify the samples by chance, we hypothesized further that better results would be obtained by restricting the number of selected features. No improvement in LOOCV misclassification rates was observed with most of the feature selection methods we examined. However, GRFS with only two features provided the best results, with a LOOCV misclassification rate of 15%. Based on additional simulations, we concluded that this rate was better than would be expected on random data without a real class structure.

We conclude that predictive models based on most current microarray data sets may have to severely limit the number of selected features in order to build models that will cross-validate and thus have a greater likelihood of generalizing to future data sets. Our results suggest that studies with 60 samples cannot use 10 features; Michiels’ results suggest that studies with 200 samples cannot use 50 features. How to determine the number of features that can be incorporated in a predictive model, as a function of the sample size, remains an open question.

The best LOOCV misclassification rate we achieved on the combined prostate cancer data set was only 15%. In addition to the small sample size (62 tumor samples), this data set is challenging for two reasons. First, because it combines data from two studies at different institutions using different microarray platforms, we expect the data to be more variable than data from a single study. Second, we tried to find a molecular predictor to distinguish low-from high-Gleason-score prostate cancer. Our models predicted the patients with low Gleason scores reasonably well, but performed poorly when identifying patients with high Gleason scores. The imbalance of the two patient population groups could contribute to the problem. However, we expect the molecular differences between these tumors to be subtler and significantly harder to detect than the molecular differences between prostate cancer and healthy prostate.

The results from this investigation suggest that we are still far from having a definitive method for feature selection. Greedy robust feature selection appears promising when linked to a strategy that limits the number of features. Due to the limited size of the combined data set, and the lack of validation through independent testing, we make no attempt to interpret the biological significance of the predictive genes identified during model development. Selecting a set of “best” predictors and building a predictive model that avoids overntting gene expression data remains a significant challenge.

## Figures and Tables

**Table 1.** Sources of Microarray Data Sets for Meta-Analysis.

Institution	Results Published	Author	Array Type	Number of Probe Sets/Clones	Healthy Samples	Tumor Samples
Harvard	Cancer Cell (2002)	Singh et al.	Affymetrix U95-Av2	12625	50	52
Stanford	PNAS (2004)	Lapointe et al.	Two-color glass cDNA	42129	41	71*

* Stanford tumor samples include 9 lymph node metastases.

**Table 2.** Summary of the Gleason Grades of Both Microarray Data Sets.

	Gleason Grade				
Institution	Low (L) 2+3, 3+2, 3+3	Medium (M) 3+4, 4+3	High (H) 4+4, 4+5	NA	Total
Harvard (52 prostate cancer patients)	19	29	4	0	52
Stanford (62 prostate cancer patients)	24	22	15	1	62
Total Patient Population	43	51	19	1	114

**Table 3.** LOOCV Results of the Prediction Models.

Model	Features Selected	LOOCV Misclassification Rate (%)
Logistic Regression - Forward Stepwise	4 – 9	31
Greedy - LDA	10 – 20	31
Genetic algorithm - LDA	10	28
PCA - LDA	10 – 20	24
Robust feature selection - LDA	5 – 11	31

**Table 4.** Results of Fitting the Model with Fewer Features.

	Misclassification Rates of Cross-validation			
Number of Features	Greedy-LDA (%)	GA-LDA (%)	PCA-LDA (%)	Robust Selection-LDA (%)
1	33	32	27	19
2	24	26	27	15
3	31	19	23	21
4	26	24	22	24
5	29	21	19	29
6	29	31	19	29
7	26	24	19	31
